# Rare and Incidental Finding of an Uninfected Urachal Cyst in an Adult Female

**DOI:** 10.1002/ccr3.70750

**Published:** 2025-08-07

**Authors:** Eisha Javed, Maheen Naveed, Umaima Naz, Shajie Ur Rehman, Javed Mehboob

**Affiliations:** ^1^ Dow Medical College Dow University of Health Sciences Karachi Pakistan; ^2^ Diagnostic Radiology Jinnah Sindh Medical University Karachi Pakistan

**Keywords:** cyst incision, dysuria, infra‐umbilical mass, percutaneous drainage, retroperitoneal abscess, urachal cyst

## Abstract

Urachal cysts are rare congenital anomalies, often asymptomatic and incidentally diagnosed, caused by abnormal persistence or incomplete obliteration of the urachus. We report a 38‐year‐old female with lower abdominal pain and UTI symptoms, incidentally found to have a non‐infected urachal cyst on CT imaging. Managed conservatively with antibiotics, the case underscores the importance of individualized treatment and vigilant follow‐up to monitor potential complications.

**Summary**

- Early detection and tailored management of urachal cysts can prevent severe complications.
- Asymptomatic cases warrant careful monitoring, while infection necessitates timely intervention.
- Surgical consultation is considered if there are any changes in cyst characteristics or clinical symptoms, which were absent in our case.

Urachal cyst is a rare congenital anomaly occurring in 1 of 5000 live births and primarily presents in males [1]. Patients with urachal cysts commonly present with symptoms including fever, abdominal pain, or tender infra‐umbilical mass; this heterogeneity of symptoms leads to misdiagnosis, which can be rectified by using ultrasonography and CT scan. Recent literature suggests a two‐phase approach involving preoperative antibiotics, initial cyst incision and drainage, followed by a complete surgical excision. Asymptomatic, non‐infected urachal cysts can be managed through observation, whereas infected urachal cysts need treatment involving antibiotics, percutaneous drainage, or surgical removal. The therapeutic option depends on the presenting signs and symptoms, as well as the patient's surgical suitability and potential surgical risks [2].

A 38‐year‐old female presented to the urology outpatient department with complaints of lower abdominal pain, dysuria, and low‐grade fever for 7 days. She described the pain to be dull and non‐radiating. On examination, her abdomen appeared soft with mild tenderness. She had no other comorbid illness. She was vitally stable but had a high WBC count of 12.5 × 10^9^/L; upon urine analysis, a few pus cells were also seen in the urine. She was diagnosed with a UTI and was initially treated with nitrofurantoin for 10 days. However, 2 months later, she returned to the clinic with the same persistent symptoms. Upon re‐evaluation, she was referred for urine analysis, which revealed an elevated leukocyte count raising concerns about pyuria. To further assess for complications, the patient was referred for a CT scan of the abdomen. This imaging was essential, as it is recommended in cases of urinary tract infection (UTI) when there is uncertainty regarding the symptoms, concerns about a complicated UTI, persistent or recurrent symptoms despite appropriate treatment, or when complications arise that require prompt intervention and management [3]. The imaging revealed a marginally enhancing hypodense area beneath the anterior abdominal wall extending up to the dome of the urinary bladder on the right side, which raised concern for a bladder or retroperitoneal abscess, complicating her ongoing UTI. This finding prompted further management, including potential drainage and a more targeted antibiotic regimen based on culture results, ensuring proper resolution of the infection and any associated complications. She was also referred for a CT scan of the abdomen, which revealed a marginally enhancing hypodense area beneath the anterior abdominal wall extending up to the dome of the urinary bladder on the right side (Figure 1). However, no fistula communication was noted with the bladder or the skin. It measured 3.2 × 7.4 × 6.5 cm in anteroposterior, transverse, and craniocaudal dimensions. The appearance suggested a urachal cyst, which was found incidentally. Given the absence of infection in the cyst, surgical intervention was deferred. The patient was prescribed appropriate antibiotics for the treatment of UTI and was scheduled for follow‐up for symptom monitoring. She was counseled about the potential complications associated with the cyst, emphasizing the importance of follow‐ups to monitor the cyst's stability. Surgical consultation was considered if there were any changes in cyst characteristics or clinical symptoms, which were absent in our case.

**FIGURE 1:**
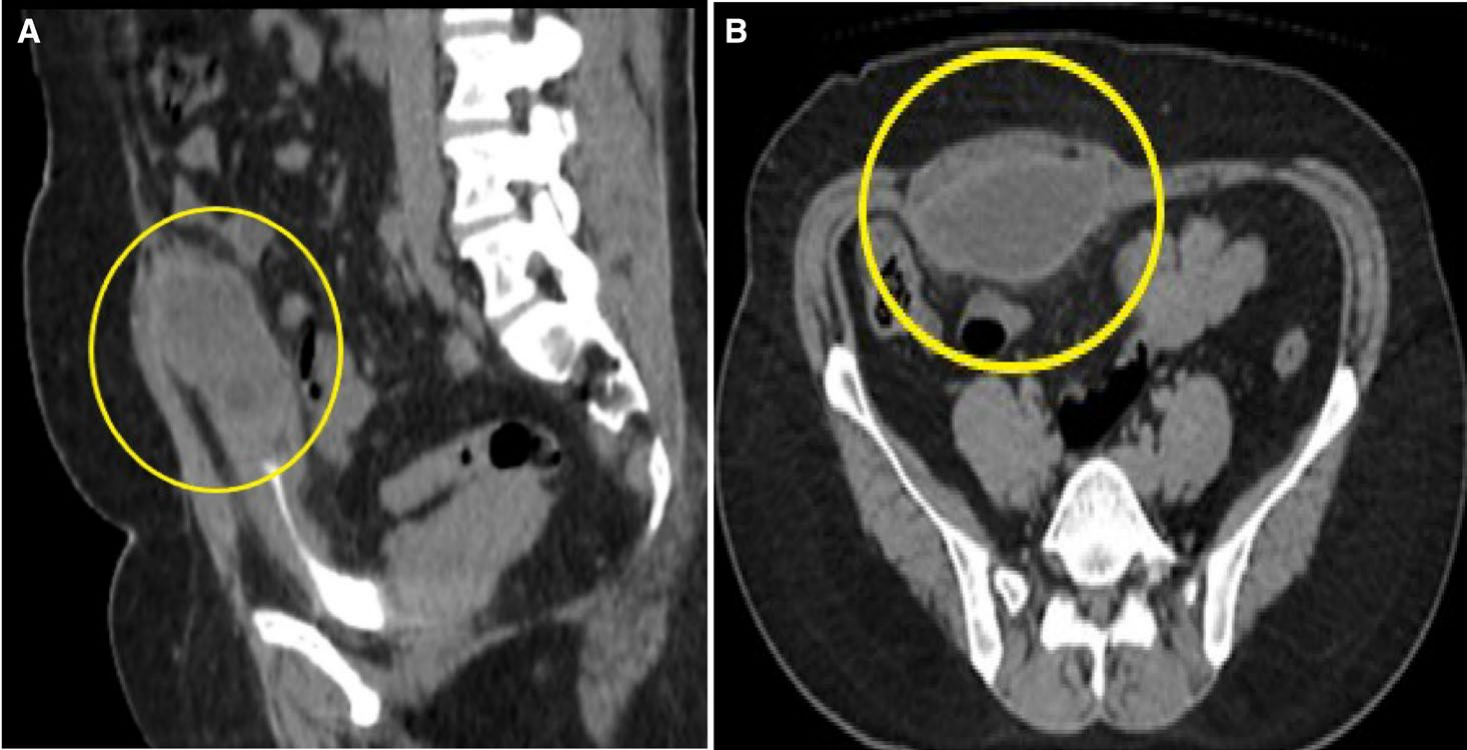
CT‐scan abdomen showing a hypodense lesion beneath the anterior abdominal wall extending up to the dome of the urinary bladder on the right side without any fistulous connection with the bladder or skin. (A) Lateral view; (B) transverse (axial) view.

## Author Contributions

**Eisha Javed:** methodology, writing – original draft. **Maheen Naveed:** methodology, writing – original draft. **Umaima Naz:** methodology, writing – original draft. **Shajie Ur Rehman:** supervision, writing – review and editing. **Javed Mehboob:** conceptualization, investigation, resources, supervision, writing – original draft, writing – review and editing.

## Consent

A written informed consent was obtained before the submission to the Journal to publish the case in accordance with the journal's patient consent policy. The patient has confirmed the same terms outlined in Wiley's standard consent form. A copy of any consent form is available from the corresponding author upon reasonable request by the publisher.

## Conflicts of Interest

The authors declare no conflicts of interest.

## Data Availability

Data sharing not applicable to this article as no datasets were generated or analyzed during the current study.
